# Enhanced Solubility of Rapeseed Meal Protein Isolates Prepared by Sequential Isoelectric Precipitation

**DOI:** 10.3390/foods9060703

**Published:** 2020-06-01

**Authors:** Hristo Kalaydzhiev, Radoslav Georgiev, Petya Ivanova, Magdalena Stoyanova, Cristina L. M. Silva, Vesela I. Chalova

**Affiliations:** 1 Department of Biochemistry and Molecular Biology, University of Food Technologies, 26 Maritsa Blvd, 4002 Plovdiv, Bulgaria; hristo.kalaydzhiev@yahoo.com (H.K.); racho95@abv.bg (R.G.); petia_ivanova_georgieva@abv.bg (P.I.); 2 Department of Analytical Chemistry and Physicochemistry, University of Food Technologies, 26 Maritsa Blvd, 4002 Plovdiv, Bulgaria; magdalena.stoianova@abv.bg; 3 CBQF-Centro de Biotecnologia e Química Fina–Laboratório Associado, Escola Superior de Biotecnologia, Universidade Católica Portuguesa, Rua Diogo Botelho 1327, 4169-005 Porto, Portugal; clsilva@porto.ucp.pt

**Keywords:** industrial rapeseed meal, sequential protein isolation, plant protein enhanced solubility

## Abstract

The solubility of plant protein isolates is a key determinant of their potential application. Two protein isolates (PI) from ethanol-treated industrial rapeseed meal, PI_10.5–2.5_ and PI_2.5–8.5_, were prepared by sequential isoelectric precipitation of alkali-extracted proteins (pH 12) starting from pH 10.5 to 2.5 or from pH 2.5 to 8.5, respectively. Biochemical analyses revealed that PI_2.5–8.5_ contained a higher amount of crude protein (72.84%) than PI_10.5–2.5_ (68.67%). In the same protein isolate, the level of total phenols (0.71%) was almost two-fold higher than that in PI_10.5–2.5_ (0.42%). No glucosinolates were established in both protein isolates. SDS-PAGE analysis demonstrated that PI_10.5–2.5_ contained 10 to 15 kDa protein fractions in a relatively higher amount, while PI_2.5–8.5_ was enriched in 18 to 29 kDa protein fractions. PI_10.5–2.5_ exhibited high solubility, varying from 41.74% at pH 4.5 to 65.13% at pH 6.5, while PI_2.5–8.5_ was almost two-fold less soluble under the same conditions. Up to pH 5.5, the addition of NaCl at 0.03 and 0.25 M diminished the solubility of PI_2.5–8.5_, while the solubility of PI_10.5–2.5_ was increased. The supplementation of PI_10.5–2.5_ with 0.25 M NaCl enhanced the protein solubility to 56.11% at pH 4.5 and 94.26% at pH 6.5. The addition of 0.03 M NaCl also increased the solubility of this protein isolate but to a lower extent. Overall, the approach for sequential precipitation of proteins influenced the biochemical characteristics, protein fractional profile and solubility of prepared protein isolates.

## 1. Introduction

Providing sufficient and high quality protein is important when the world’s population is expected to grow to almost 10 billion by 2050 [1]. Sociodemographic change, as well as the global increase of middle classes, drives the food industry to the production of new proteins by efficient utilization of natural resources and gentle care of the environment [2]. Plant proteins are abundant and less expensive alternatives to animal proteins [3]. Among them, rapeseed meal proteins are considered prospective nutritive and functional ingredients for the food industry [4,5]. The most common approach to prepare protein-rich ingredients, namely protein isolates (PI), is by isoelectric precipitation after extraction with NaOH [6,7,8,9]. The precipitation occurs at a single pH where the solubility of the proteins is the lowest which, however, diminishes their potential practical application. The solubility of rapeseed protein isolates is a major determinant of functional properties such as water and oil absorption capacity, gelling, foaming and emulsification [10,11]. The solubility of the rapeseed protein isolates, being used as additives in food processing, has a strong influence on protein dispersion and physicochemical properties of the food colloidal system. This property is highly dependent on protein isolate constituents, major and minor protein components, their structures, degree of association and transition states under conditions used for their preparation [4]. In many cases, chemical, physical or enzymatic modifications are applied to enhance the solubility, and as a consequence, expand the range of their functionality [6,10,11]. These procedures, however, might be costly, complicated and financially inefficient.

So far, there is no agreement about the isoelectric point of the rapeseed proteins. El Nockrashy et al. [12] and Pedroche et al. [6] observed the lowest solubility of the proteins in two pH ranges, one in the highly acidic region, pH 3.5 to 3.6, and the other in the slightly acidic region, pH 5 to 6. Ghodsvali et al. [13] reported that the isoelectric point of the rapeseed proteins was in the range from pH 4.5 to 5.5. Lönnerdal and Janson [14] found that 20% to 40% of rapeseed proteins had an isoelectric point close to pH 11, while for the rest of the proteins it was in the range of 4 to 8. Since different fractions of proteins precipitate at different pH values, the corresponding isolates are expected to have different biochemical characteristics and potential practical applications.

This study aimed to evaluate the influence of sequential isoelectric precipitation methodology on biochemical characteristics and solubility of rapeseed meal protein isolates. While most investigations are focused on canola or laboratory prepared rapeseed meals, in this study, industrially produced rapeseed meal was used as a protein-rich source. The industrial production of the meal differs by relatively higher temperatures and the involvement of chemical reagents, which might worsen the properties of the proteins, including their solubility. Although challenging, studies on utilization of rapeseed meals, produced under industrial conditions, for production of ingredients with an added value are of greater practical application and importance.

## 2. Materials and Methods

### 2.1. Preparation of Protein Isolates

Industrially manufactured rapeseed meal was obtained from a local company. It was ground and sieved to obtain particles with a uniform size (≤0.315 mm). To reduce the content of phenols and glucosinolates, the material was subjected to a 4-step treatment with a 75% aqueous ethanol solution (*v*/*v*) at a meal-to-solvent ratio of 25% (*w*/*v*) for 30 min at room temperature [11]. The residue was collected by decanting, then dried in air and stored in a closed container for further use as a source for preparation of protein isolates. Proteins were extracted from a 5% (*w*/*v*) ethanol-treated rapeseed meal suspension (pH 12) at 40 °C for 60 min under continuous agitation. Two protein isolates were prepared from the extract. The first protein isolate PI_0.5–2.5_ was obtained by sequential precipitation of the proteins from the extract starting at pH 10.5, followed by lowering the pH by 1 unit to 2.5 with HCl. The precipitate, obtained at each pH value, was collected by centrifugation for 15 min at 1800× *g* (MPW-251, Med. Instruments, Warszawa, Poland) and the supernatant was used for isoelectric precipitation at the following pH value. The second protein isolate, PI_2.5–8.5_, was obtained after a sharp decrease of extract pH to 2.5. Further, precipitates were obtained by sequential isoelectric precipitation by increasing the pH to 8.5 with an increment of 1. The sediments, obtained at each pH value, were dried by lyophilization (Lyovac GT2, Leybold Heraeus, Köln, Germany), mixed and homogenized to prepare PI_10.5–2.5_ or PI_2.5–8.5_.

### 2.2. Chemical Analyses

Protein isolates were chemically characterized by using well established and standard methods. Crude protein was determined by an AOAC official method [15]. The coefficient 6.25 was used to convert total nitrogen to crude protein. Ash content was determined by ICC Standard №104/1 [16]. The amounts of total lipids and carbohydrates were evaluated as described by Bligh and Dyer [17] and Dubois et al. [18]. Total phenols were extracted with 70% aqueous ethanol solution [19] and quantified by using Folin–Ciocalteu reagent [20]. Total glucosinolates were evaluated as described by Jezek et al. [21]. The method is based on spectrophotometric evaluation of glucosinolates after alkaline hydrolysis and reduction with potassium ferricyanide. Sinigrin was used for standard curve generation. Selenium (Se) was determined by using inductively coupled plasma optical emission spectrometry (ICP-OES) [22]. For all other microelements and heavy metals, Bulgarian National Standard procedure BDS 11374 [23] was used.

### 2.3. Amino Acid Analyses and Amino Acid Score Calculation

Samples were hydrolyzed with 6NHCl at 105 °C for 24 h followed by neutralization and filtration [24]. The hydrolysates were derivatized by using the AccQ-Fluor TM Reagent kit (Waters Corporation, Milford, MA, USA) following the manufacturer’s instructions. The amino acid analyses were performed by high performance liquid chromatography (ELITE La Chrome, Hitachi High Technologies America, Inc., San Jose, CA, USA) equipped with a C18AccQ-Tag (3.9 × 150 mm) reversed-phase chromatographic column and a diode array detector. Amino acid score (AAS) was calculated as a ratio of the amount of each essential amino acid in a sample (g/100 g protein) and the amount of the respective amino acid in an “ideal” protein (g/100 g protein) as defined by the World Health Organization [25]. The results were multiplied by 100 to express in percentage.

### 2.4. Sodium Dodecyl Sulfate Polyacrylamide Gel Electrophoresis (SDS-PAGE)

SDS-PAGE was performed under reducing conditions with an omniPAGE mini Cleaver electrophoresis (model CVS10DSYS, Cleaver Scientific Ltd., Rugby, UK) as described by Laemmli [26]. The gel system consisted of a 15% polyacrylamide resolving gel (pH 8.8). The gel was immersed in a 0.2% Coomassie Brilliant Blue R-250 dye (Serva Electrophoresis GmbH, Heidelberg, Germany) solution for 20 min for visualization. Discoloration of the gel was achieved by a solution containing 10% ethanol and 7% acetate overnight. Data were analyzed by using TotalLab1D Analysis software (BioStep GmbH, Burkhardtsdorf, Germany). To ease readability and data analyses, proteins were provisionally grouped into three categories: low molecular weight (LMW) proteins with a molecular weight up to 50 kDa, medium molecular weight (MMW) proteins with a molecular weight ranging from 50 to 150 kDa and high molecular weight (HMW) proteins with a molecular weight above 150 kDa.

### 2.5. Protein Solubility

Protein solubility of PI_10.5–2.5_ or PI_2.5–8.5_ was determined as previously described [9]. Samples were dispersed in water to a final protein concentration of 4 mg/mL. The pH varied from 2 to 8.5 with an increment of 0.5 by using either NaOH or HCl. NaCl was added to a final concentration of 0.03 or 0.25 M where appropriate. After 2 h at room temperature, samples were centrifuged for 15 min at 1800× *g* (MPW-251, Med. Instruments, Poland) and supernatants were collected. Protein solubility was calculated as a ratio of the amount of the protein in a supernatant, determined by the Biuret method [27], and the crude protein content of the sample used in the analysis. The result was multiplied by 100 to express in percentage. Bovine serum albumin was used as a standard protein.

### 2.6. Statistical Analyses

Except for amino acids, analyses were performed in triplicates. Results are presented as means ± standard deviation (SD). Data were analyzed by one-way analysis of variance (ANOVA) using the Statgraphics Centurion statistical program (version XVI, 2009) (Stat Point Technologies, Ins., Warrenton, VA, USA). Mean differences were established by Fisher’s least significant difference test for paired comparison with a significance level α = 0.05.

## 3. Results and Discussion

### 3.1. Biochemical Characterization of PI_10.5–2.5_ and PI_2.5–8.5_

The application of the two approaches resulted in the generation of protein isolates in different amounts. Starting with 100 g ethanol-treated rapeseed meal, containing 42.25% protein (dry matter basis), 15.60 g PI_10.5–2.5_ and 11.76 g PI_2.5–8.5_ were obtained. The latter, although obtained in lower quantity, was characterized with higher purity. The biochemical analyses revealed that PI_2.5–8.5_ contained a higher amount of crude protein (72.84%) than PI_10.5–2.5_ (68.67%) (Table 1). Compared to a rapeseed meal protein isolate, previously prepared by precipitation at pH 4.5 [28], both isolates contained higher amounts of concomitant compounds. Isoelectric precipitation is a common approach used for alkali-extracted plant protein isolation and preparation of protein-rich products with relatively high purity. Following different extraction conditions, Pedroche et al. [6] were able to prepare rapeseed protein isolates with protein content higher than 90%. In other studies, the protein content of the generated rapeseed/canola meal protein isolates varied from 70% to 90% [13,29].

PI_2.5–8.5_ contained an almost two-fold higher amount of phenols (0.71%) than PI_10.5–2.5_ (0.42%) (Table 1). In most cases, the interaction of proteins with phenols is mediated by ionic and hydrogen bonds, and therefore, it is highly influenced by ionic strength and pH [30]. In addition, it was demonstrated that the effect is strongly differential and is dependent on the type of phenolic compounds and proteins. By studying the interactions of pure phenolics (gallic acid, ferulic acid, chlorogenic acid, quercetin, apigenin, catechin) and phenolics from plant extracts (green tea, green coffee) with white bean proteins, albumins and globulins, the same authors established that all protein–phenol complexes had a negligible solubility in pH ranging from 4 to 5. This might be the reason for the higher amount of phenols observed in PI_2.5–8.5_ than in PI_10.5–2.5_. Considering the lack of more detailed information about phenol and protein composition of the isolates, a more precise conclusion should be made after additional investigation of the samples.

Rapeseed meal is rich in phenols and other bioactive compounds which could be used for the preparation of functional foods [31]. In general, phenols are considered to have health-beneficial properties. Being a part of protein isolates though, they could worsen nutritive and functional properties of the proteins [32]. To reduce their content in the final products, an ethanol-treated rapeseed meal was used as a protein source for the preparation of the isolates. A previous study by Kalaydzhiev et al. [33] demonstrated a four-fold reduction of phenolic content in the rapeseed meal after a four-step treatment with 75% aqueous ethanol solution. Still, by using the sequential precipitation technique, the total phenolic levels in both protein isolates prepared, 0.42% for PI_10.5–2.5_ and 0.71% for PI_2.5–8.5_ (Table 1), exceeded the one in the single pH rapeseed meal protein precipitate previously established (0.26%) [28]. No glucosinolates were found in both protein isolates.

### 3.2. Electrophoretic Protein Profile of PI_10.5–2.5_ and PI_2.5–8.5_

**Figure 1:**
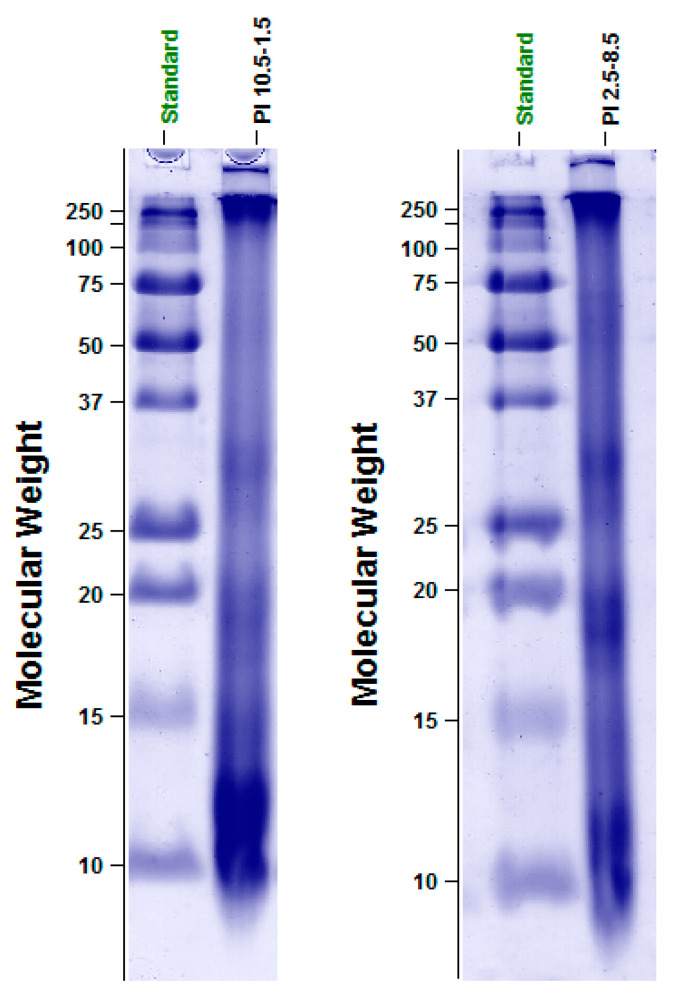
SDS-PAGE of PI_10.5–2.5_ and PI_2.5–8.5_. PI: protein isolates.

**Figure 2:**
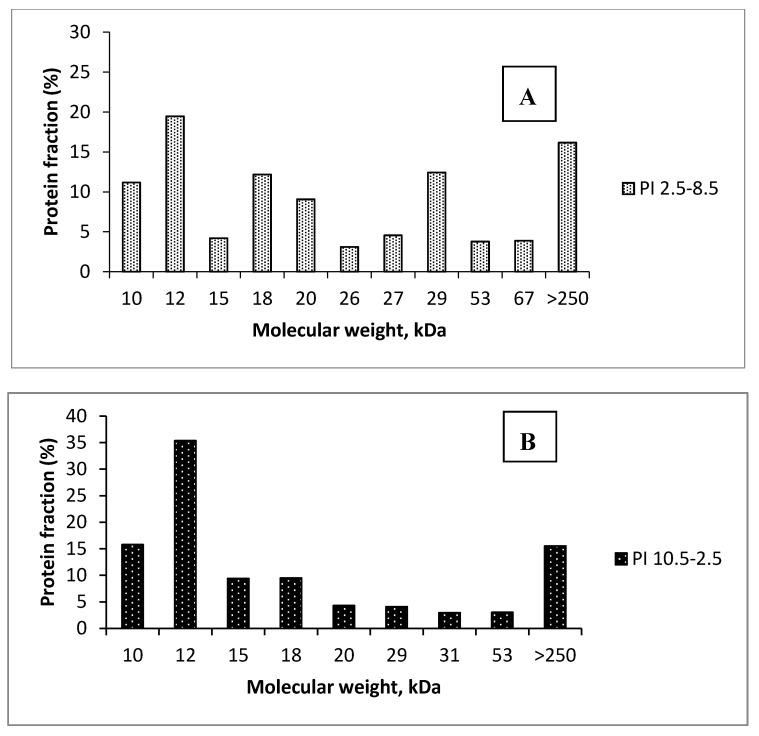
Fractional profiles of PI_2.5–8.5_ (**A**) and PI_10.5–2.5_ (**B**).

SDS-PAGE analysis revealed differences in the protein profile of the two protein isolates (Figure 1). They were more noticeable in the group of LMW proteins (molecular weight up to 50 kDa) where protein fractions differed in both composition and quantity (Figure 2A,B). Two LMW fractions, 12 and 15 kDa, were established in almost two-fold higher amounts in PI_10.5–2.5_ (35.36% and 9.42%) than in PI_2.5–8.5_ (19.48% and 4.19%). A protein with a molecular weight of 10 kDa was established in PI_10.5–2.5_ in a higher amount (15.79%) than in PI_2.5–8.5_ (11.17%). Most probably, they belong to 2S-albumins which are major storage proteins in rapeseed. Depending on rapeseed varieties, their amount may vary from 20% to 40% from aqueous soluble rapeseed protein [34]. A study by Monsalve and Rodriguez [35] suggested the existence of eleven 2S-albumin proteins with high similarity in structure and molecular weights ranging from 12.5 to 15 kDa. By using SDS-PAGE analysis, Höglund et al. [36] observed two polypeptide chains derived from napin (a 2S-albumin), one having molecular weight of 14 kDa, and the other, a lighter one, with a molecular weight close to 10 kDa. A band, corresponding to 9.5 and 9.0 kDa, was attributed to napin by Wu and Muir [37] and Adem et al. [38] (2014), respectively. The enhanced relative amount of the protein fractions with molecular weight from 10 to 15 kDa in PI_10.5–2.5_ could be attributed to the basic nature of 2S-albumins [39]. They have isoelectric point (pIs), and respectively lowest solubility, in a pH range from 10 to 11 where the isoelectric precipitation for the preparation of PI_10.5–2.5_ was initiated. However, some napin isoforms may differ in their pI, and therefore, precipitate at different pH values, lower than 10 [39].

A band corresponding to 20 kDa protein was observed in the SDS-PAGE profile of both protein isolates (Figure 1). The electrophoretic analysis revealed that the 20 kDa protein fraction was in a relatively higher amount in PI_2.5–8.5_ than in PI_10.5–2.5_ (Figure 2A,B). The latter contained 4.31% of that protein fraction, while its amount in PI_2.5–8.5_ was almost doubled and reached 9.07%. Since SDS-PAGE was performed under reducing conditions, observed electrophoretic bands may correspond to either monomeric proteins or protein subunits. The 20 kDa protein fraction, observed in this investigation, might belong to the group of oleosins which is the third most studied group of rapeseed proteins. Oleosines are membrane proteins with a molecular weight at around 20 kDa and represent approximately 6% to 8% of the rapeseed proteins [39]. As having an isoelectric point of pH 6.5 [40], their precipitation was favored by that starting at 2.5. On the other hand, a band corresponding to 20.1 kDa protein was claimed by Höglund et al. [36] as the β-chain of cruciferin. Rapeseed cruciferin is a hexamer which exhibits reversible association and dissociation depending on pH and ionic strength as the mature protein has a pI at pH 7 [39]. The same author reported that cruciferin subunits separated in SDS-PAGE under reducing conditions and formed several bands in the range of 18 to 53 kDa. Perera et al. [41] also indicated that the crusiferin molecule resolved under reducing conditions as single bands between 14.0 and 32.0 kDa, while napin was observed as two bands at around 10 and 14 kDa. It should be noted that all protein fractions from 18 to 29 kDa were in higher amounts in PI_2.5–8.5_ than in PI_10.5–2.5_ (Figure 2A,B).

In a small amount (3.89%), a 67 kDa protein was established in PI_2.5–8.5_ but not in PI_10.5–2.5_. By analyzing a rapeseed meal protein isolate, prepared by isoelectric precipitation at 4.5, Ivanova et al. [28] observed a minor band corresponding to a 66 kDa protein. A similar protein was reported by Adem et al. [38], who studied the SDS-PAGE protein profile of a rapeseed protein concentrate. In both studies, the nature of that protein was not established. HMW proteins, as established by SDS-PAGE, were presented in relatively small amounts (9–10, Table 2). Our data agreed with Perera et al. [41], observing that rapeseed proteins, soluble at pH 12, showed disappearance of polypeptide bands in the medium and high molecular weight region (molecular weight >50 kDa), while polypeptides between 10 and 34 kDa were easily identifiable.

Based on the results from SDS-PAGE analyses, we hypothesize that sequential isoelectric precipitation from pH 10.5 to 2.5 favored the isolation of basic proteins and could be used for the preparation of protein isolates enriched in these types of proteins. In contrast, sequential precipitation of the proteins from pH 2.5 to 8.5 facilitated the isolation of proteins with pIs in the acidic pH area and enhanced their amounts in PI_2.5–8.5_. The assumption is based on the relative prevalence of specific fractions which, according to data available in the literature, are acquired from proteins with pIs either in the alkaline or acidic pH range. Still, more precise experiments involving model systems with known proteins need to be performed for a more confirmative conclusion.

### 3.3. Amino Acid and Microelemental Composition of PI_10.5–2.5_ and PI_2.5–8.5_ Protein Isolates

Essential and non-essential amino acid compositions of PI_2.5–8.5_ and PI_10.5–2.5_ are presented in Table 3 and Table 4, respectively. The amino acid profiles of the two isolates differed in the amounts of most of the amino acids analyzed. The contents of all essential amino acids in PI_10.5–2.5_ exceeded that in PI_2.5–8.5_ (Table 3). Except for the isoleucine, their AASs were higher than 100%, meaning higher contents of these amino acids compared to the ones in a reference protein [25]. The most profound differences were observed in the levels of valine and lysine. In PI_10.5–2.5_, they were higher than that in PI_2.5–8.5_ with approximately 2 and 3 g/100 g protein, respectively (Table 3). Following the assumption that PI_10.5–2.5_ is richer in napin (albumin) and other basic proteins, our results agreed with the data published by Chabanon et al. [11], where lysine and valine were detected in higher amounts in the albumin fraction than in the globulin one. The combined amounts of phenylalanine and tyrosine, and methionine and cysteine also exceeded the ones of the reference protein [25]. While data on amino acid composition demonstrate the sufficiency of most of the essential amino acids, additional investigation of the digestibility of both protein isolates is needed to evaluate their nutritive potential.

Arginine, which, similarly to lysine, has a basic side chain, was in a higher amount in PI_10.5–2.5_ (4.93 g/100 g protein) than in PI_2.5–8.5_ (3.92 g/100 g protein) as well (Table 4). On the other hand, aspartate was four-fold higher in PI_2.5–8.5_ (18.03 g/100 g protein) than in PI_10.5–2.5_ (4.36 g/100 g protein).

Copper, Fe, Zn, Mn and Se are essential trace elements which have important biological functions in cells. They are considered to contribute to structure stabilization of macromolecules and enzyme activity implementation [42]. Lack or insufficiency of any of these elements may result in serious illnesses or physiological discomfort [43]. In the current study, PI_2.5–8.5_ and PI_10.5–2.5_ exhibited differences in the content of the evaluated microelements (Table 5). While Fe and Mn were in higher amounts in PI_10.5–2.5_, PI_2.5–8.5_ was richer in Cu and Zn. Most probably, these observations are due to differences in the protein composition of the isolates, the extent of their interaction with microelements and the stability of the respective complexes at the pH of isoelectric isolation but were not completely understood. In contrast, Se content in the two protein isolates was unexpectedly below the detection minimum of the method (Table 5). In general, plant-derived foods are poor in Se but the content of this microelement is relatively high in rapeseed meals, varying from 0.16 to 0.29 mg/kg [44]. In a previous study, Ivanova et al. [28] established 1.07 and 0.87 mg/kg Se in the protein isolate and acid soluble protein prepared from industrial rapeseed meal, respectively. The same authors reported high levels of Pb (1.48 mg/kg) and Cd (0.10 mg/kg) in the rapeseed protein isolate as well, while in the current study no heavy metals were established in the two products. *Brassica* plants are well known for their ability to accumulate heavy metals from soil [45]. However, their presence (if at all) and content in rapeseed-derived products is highly variable and depends on *Brassica* cultivars metal uptake capacity and soil contamination [45,46]. Therefore, the content of heavy metals in rapeseed-derived products needs to be evaluated prior to food application.

### 3.4. Solubility of PI_10.5–2.5_ and PI_2.5–8.5_ Protein

**Figure 3:**
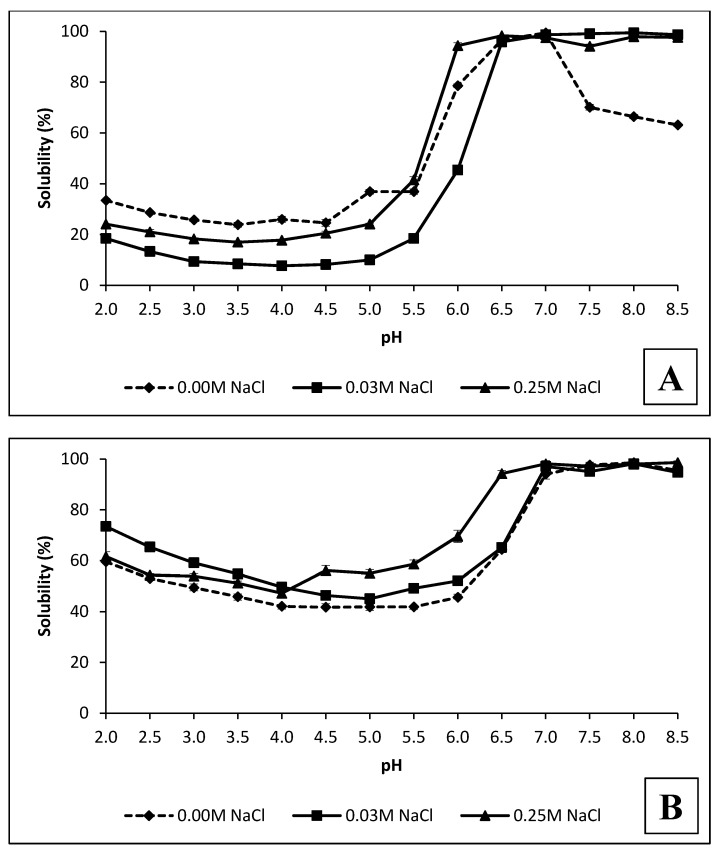
Solubility of protein isolates PI_2.5–8.5_ (**А**) and PI_10.5–2.5_ (**B**) at different pH values and NaCl concentrations.

The solubility of PI_2.5–8.5_ and PI_10.5–2.5_ is presented in Figure 3A,B. It was explored in the presence of two levels of NaCl concentrations, 0.03 and 0.25 M, and a broad pH area ranging from 2 to 8.5. Among the numerous factors influencing protein solubility, pH and NaCl supplementation are the ones having the highest practical impact [4,39]. The two protein isolates exhibited a similar pattern of water solubility, namely almost complete solubility of the protein at pH ≥7 and lower solubility below that point. However, they differed significantly in the extent of solubility in the acidic pH area. PI_10.5–2.5_ exhibited high solubility, varying from 41.74% at pH 4.5 to 65.13% at pH 6.5 (Figure 3B). PI_2.5–8.5_ was almost two-fold less soluble under the same conditions. Most probably, this is due to the differences established in the fractional profiles of the PI_2.5–8.5_ and PI_10.5–2.5_ proteins. PI_10.5–2.5_ presumably contained basic proteins in relatively higher amounts. Having isoelectric points in the alkaline area (pH 9–10.5), these protein molecules are supposed to be charged positively in acidic pH and as a consequence, better hydrated. Jiang et al. [47] suggested that an elevated surface charge is needed to provide sufficient electrostatic repulsion and maintain the molecules in solution. According to Wanasundara et al. [4], canola proteins that remain insoluble at an acidic pH range are predominantly cruciferin, while napin has a relatively high solubility in a wider pH range from 2 to 10. Most probably, basic protein availability in PI_10.5–2.5_ is contributing to the better solubility of this protein isolate compared to PI_2.5–8.5_. The latter, as prepared by sequential precipitation from pH 2.5 to 8.5, has a relatively higher amount of proteins with isoelectric points in this acidic area, and as a consequence, has a lower solubility in that pH range. This assumption is supported by electrophoretic profiles of both isolates where higher relative amounts of protein fractions of 2S-albumins and cruciferin, typically observed under reducing conditions, were established in PI_10.5–2.5_ and PI_2.5–8.5_, respectively. Although lower than that of PI_10.5–2.5_, the solubility of PI_2.5–8.5_ is still almost 10-fold higher than the negligible solubility (2.80%) of the rapeseed meal protein isolate prepared by isoelectric precipitation at pH 4.5 [9].

The solubility of both protein isolates, PI_2.5–8.5_ and PI_10.5–2.5_, was influenced by the addition of NaCl but in a different way (Figure 3A,B). Up to pH 5.5, the addition of NaCl at the two levels diminished the solubility of the PI_2.5–8.5_, while the solubility of PI_10.5–2.5_ was increased. The supplementation of PI_10.5–2.5_ with 0.25 M NaCl enhanced the protein solubility to 56.11% at pH 4.5 and 60.55% at pH 6.0. The addition of NaCl at a low concentration (0.03 M) also increased the solubility of this protein isolate but to a lower extent. In general, the addition of NaCl in low concentrations enhances protein solubility. The positive effect of the salts is due to the electrostatic interactions between the salt ions and the charges in the protein molecule [48]. Proteins are complex polyelectrolytes having positively and negatively charged regions. In the presence of low concentrations of salts, the protein molecule is surrounded by an excess of oppositely charged ions, thereby reducing electrostatic interactions between the proteins. As a result, their solubility is increased. However, taking into account compositional protein variation of PI_2.5–8.5_ and PI_10.5–2.5_, the salt-specific effect may need to be considered as well. The affinity of ions to hydration is presented by the Hofmeister series [49]. A recent study indicated that not only are hydration properties of ions important, but also their interaction with protein surface groups should be considered and understood to better explain their effect on protein solubility [50]. This becomes even more complex since the relative effectiveness of different anions on protein electrostatic interactions is pH-dependent and follows a reverse Hofmeister sequence for pH < pI and a direct Hofmeister sequence for pH > pI [49]. Due to the high variability of proteins composing PI_2.5–8.5_ and PI_10.5–2.5_, and potential interaction with NaCl and non-protein compounds, the effect of salt supplementation on solubility is not straightforward and more profound investigations are needed to better understand the mechanism.

## 4. Conclusions

The solubility of the rapeseed protein isolates, being used as additives in food processing, has a strong influence on protein dispersion and physicochemical properties of food colloidal systems. The two approaches for sequential precipitation of alkali-extracted plant proteins resulted in protein-rich products that differed in biochemical characteristics and fractional profiles. The variability in protein fractional composition and relative content of both protein isolates affected their solubility. The latter was dependent on both pH and NaCl supplementation. PI_10.5–2.5_ and PI_2.5–8.5_ exhibited almost complete protein solubility at pH ≥ 7 and lower solubility below that point. The two protein isolates, however, differed significantly in the extent of solubility in the acidic pH area. PI_10.5–2.5_ exhibited high solubility varying from 41.74% at pH 4.5 to 65.13% at pH 6.5. PI_2.5–8.5_ was almost two-fold less soluble under the same conditions. Therefore, sequential precipitation of proteins could be used to prepare protein isolates with desired protein profiles, enhanced solubility and potentially improved application. Additional investigation on their gelling capacity, foaming and emulsification properties would reveal and better outline the possible use of those rapeseed protein meal isolates as alternatives to protein additives currently applied in food processing.

## Figures and Tables

**Table 1:** Chemical composition of protein isolates (PI), PI_10.5–2.5_ and PI_2.5–8.5_.

Component	Content * (%)	
	PI_10.5–2.5_	PI_2.5–8.5_
Crude protein	68.67 ± 0.16 ^b^	72.84 ± 0.45 ^a^
Ash	13.18 ± 0.73 ^a^	10.45 ± 0.11 ^b^
Total lipids	2.85 ± 0.14 ^a^	2.83 ± 0.03 ^а^
Total carbohydrates	4.02 ± 0.10 ^а^	3.76 ± 0.30 ^а^
Total phenols	0.42 ± 0.01 ^b^	0.71 ± 0.05 ^a^
Glucosinolates	ND	ND

* Calculated on a dry matter basis of protein isolates, 94.68% ± 0.05% for PI_10.5–2.5_ and 97.39% ± 0.02% for PI_2.5–8.5_. ^a,b^ Means in a row with different superscripts differ significantly (*p* < 0.05). ND: not detected.

**Table 2:** Protein fraction distribution of PI_2.5–8.5_ and PI_10.5–2.5_.

Proteins	Protein Distribution (%)	
	PI_2.5–8.5_	PI_10.5–2.5_
LMW	81.8	77.8
MMW	9.1	11.1
HMW	9.1	11.1

LMW: proteins with molecular weights <50 kDa; MMW: proteins with molecular weights from 50 to 150 kDa; HMW: proteins with molecular weights >150 kDa.

**Table 3:** Essential amino acid composition and amino acid score of PI_10.5–2.5_ and PI_2.5–8.5_.

Amino Acid	Reference Protein * (g/100 g Protein)	PI_2.5–8.5_		PI_10.5–2.5_	
		Content (g/100 g Protein)	Amino Acid Score (%)	Content (g/100 g Protein)	Amino Acid Score (%)
Valine	3.9	3.99 ± 0.14	102.30	5.95 ± 0.20	152.56
Leucine	5.9	5.26 ± 0.90	89.15	6.74 ± 0.16	114.23
Isoleucine	3.0	1.96 ± 0.30	65.36	2.25 ± 0.18	75.00
Threonine	2.3	2.66 ± 0.15	115.65	2.99 ± 0.13	130.00
Lysine	4.5	4.51 ± 0.22	100.22	7.55 ± 0.11	167.77
Phenylalanine+ tyrosine	3.8	4.72 ± 0.27	ND	4.82 ± 0.94	ND
Methionine+ cysteine	2.2	3.95 ± 0.25	ND	3.99 ± 0.13	ND

* Amino acid composition of a reference protein [25]; ND: not determined.

**Table 4:** Non-essential amino acids composition of PI_10.5–2.5_ and PI_2.5–8.5_.

Amino Acid	PI_2.5–8.5_	PI_10.5–2.5_
	Content (g/100 g Protein)	Content (g/100 g Protein)
Alanine	5.64 ± 0.11	7.38 ± 0.15
Glycine	2.05 ± 0.27	2.54 ± 0.19
Arginine	3.92 ± 0.15	4.93 ± 0.24
Serine	3.07 ± 0.10	1.52 ± 0.27
Aspartate	18.03 ± 0.17	4.36 ± 0.18
Glutamate	10.55 ± 0.16	11.79 ± 0.12
Histidine	1.75 ± 0.09	1.39 ± 0.17
Proline	3.77 ± 0.10	5.22 ± 0.24

**Table 5:** Selected microelements and heavy metal contents of PI_2.5–8.5_ and PI_10.5–2.5_.

Component	Content * (mg/kg)	
	PI_2.5–8.5_	PI_10.5–2.5_
Copper (Cu)	28.85 ± 0.02 ^a^	18.63 ± 0.06 ^b^
Iron (Fe)	178.81 ± 0.37 ^b^	301.53 ± 16.28 ^a^
Manganese (Mn)	129.15 ± 4.03 ^b^	143.17 ± 1.43 ^a^
Selenium (Se)	<0.1	<0.1
Zinc (Zn)	137.25 ± 8.83 ^a^	119.90 ± 1.36 ^b^
Lead (Pb)	<0.1	<0.1
Cadmium (Cd)	<0.01	<0.01

* Calculated on a dry matter basis, 94.68% ± 0.05% for PI_10.5–2.5_ and 97.39% ± 0.02% for PI_2.5–8.5_. ^a,b^ Means in a row with different superscripts differ significantly (*p* < 0.05).
